# Microstructure, Physical Properties, and Oxidative Stability of Olive Oil Oleogels Composed of Sunflower Wax and Monoglycerides

**DOI:** 10.3390/gels10030195

**Published:** 2024-03-13

**Authors:** Dafni Dimakopoulou-Papazoglou, Konstantina Zampouni, Prodromos Prodromidis, Thomas Moschakis, Eugenios Katsanidis

**Affiliations:** Department of Food Science and Technology, School of Agriculture, Faculty of Agriculture, Forestry and Natural Environment, Aristotle University of Thessaloniki, 54124 Thessaloniki, Greece; ddimakop@agro.auth.gr (D.D.-P.); zampounikona@gmail.com (K.Z.); prodromosprod@gmail.com (P.P.); tmoschak@agro.auth.gr (T.M.)

**Keywords:** oleogels, sunflower wax, lipid structuring, crystallization, FTIR, storage

## Abstract

The utilization of natural waxes to form oleogels has emerged as a new and efficient technique for structuring liquid edible oil into solid-like structures for diverse food applications. The objective of this study was to investigate the interaction between sunflower wax (SW) and monoglycerides (MGs) in olive oil oleogels and assess their physical characteristics and storage stability. To achieve this, pure SW and a combination of SW with MGs in a 1:1 ratio were examined within a total concentration range of 6–12% *w*/*w*. The formed oleogels were characterized based on their microstructure, melting and crystallization properties, textural characteristics, and oxidative stability during storage. All the oleogels were self-standing, and, as the concentration increased, the hardness of the oleogels also increased. The crystals of SW oleogels were long needle-like, while the combination of SW and MGs led to the formation of crystal aggregates and rosette-like crystals. Differential scanning calorimetry and FTIR showed that the addition of MGs led to different crystal structures. The oxidation results revealed that oleogels had low peroxide and TBARS values throughout the 28-day storage period. These results provide useful insights about the utilization of SW and MGs oleogels for potential applications in the food industry.

## 1. Introduction

Solid fats are essential components of food products as they significantly contribute to their technological and organoleptic characteristics, aligning with consumers’ preferences for food structure, aroma, and taste [1,2]. Nevertheless, their use in food products should be limited in accordance with the recommendations of the World Health Organization [3] and the Food and Agriculture Organization of the United Nations (FAO) [4], which advocate for reducing the intake of saturated fatty acids due to their association with an increased risk of various diseases [1,5]. An alternative approach that has garnered attention in recent years involves the use of oleogels as a substitute for traditional fats and for the delivery of bioactive compounds [6,7,8]. Oleogels are formed by entrapping liquid vegetable oils within a three-dimensional network using small amounts of one or more gelators, resulting in structures which mimic the properties of solid fats [9,10,11,12]. Specifically, vegetable oil, often with a high nutritional value, and structuring agents undergo heating to a temperature exceeding the melting point of the gelators. As the mixture subsequently cools, a crystal network forms, establishing a self-assembling system which traps the liquid phase through the creation of weak interactions, such as hydrogen bonds, van der Waals forces, and electrostatic interactions [13,14]. Various substances have been successfully employed as gelators to form oleogels, including mono- and diglycerides, fatty acids, fatty alcohols, phytosterols, waxes, etc. [15,16,17].

Monoglycerides (MGs) have been extensively studied for their satisfactory gelling ability and their physicochemical and structural properties that render them appropriate for numerous food applications [6,18,19]. The characteristics of the produced MGs oleogels are impacted by factors including their concentration, the oil type, and the processing conditions [1]. Nevertheless, a concentration exceeding 10% *w*/*w* is deemed appropriate for establishing a crystalline network that traps the oil [19]. Therefore, in order to both decrease the MGs concentration and enhance the stability and physicochemical properties of oleogels, MGs can be combined with other structurants, such as phytosterols [8,20,21], waxes [22,23,24,25], etc. Waxes are another type of structurants that can be effective at forming crystalline structures at low concentrations [26]. Several natural waxes have been studied in the literature, including carnauba wax [27,28], rice bran wax [26,29], candelilla wax [30,31], sunflower wax [25,32], and beeswax [33,34]. Typically, waxes consist of a variety of chemical constituents, such as long-chain fatty alcohols, long-chain fatty acids, straight-chain alkanes, and wax esters [35]. The individual components in waxes differ both in concentration and in ingredients according to their type. Waxes that are rich in wax esters and fatty alcohols create oleogels with a higher hardness [25,36]. Even though wax-based oleogels result in desirable physical properties and oxidative stability compared to MG-based oleogels [37], they lead to an unpleasant waxy mouthfeel, limiting their use [38]. 

Several waxes, including rice bran wax, candelilla wax, carnauba wax, and beeswax, have been recognized as being safe (GRAS) by the FDA [39,40]. Sunflower wax has not yet been deemed to be GRAS; however, it is considered non-toxic [41] and is naturally present, in a small percentage, in sunflower seeds, which are consumed by humans. The potential utilization of oleogels, composed of waxes like candelilla, rice bran, beeswax, and sunflower wax, in edible oils in order to replace the solid fat component has been explored for various food applications, including meat products (such as fermented sausages, emulsion-type sausages, burgers, and meat patties), dairy products (ice cream, cheeses, etc.), confectionery items (cookies, cakes, bread, etc.), chocolate, and spreadable products (margarine, fillings, creams, toppings, mayonnaise) [7,42]. According to well-documented reviews [7,42], sunflower wax has been investigated in various foods, such as cookies, sponge cakes, margarines, spreads, and sucuk sausage. 

A combination of gelators, e.g., waxes and MGs, can form oleogels with desirable physicochemical characteristics while reducing the unpleasant waxy sensation in the mouth and, at the same time, minimizing the total concentration of structurants. Barroso et al. [23] concluded that a 1:1 ratio of MGs and sunflower wax (SW) in flaxseed oil resulted in improved properties of the oleogels. Pakseresht et al. [36] studied a combination of MGs with carnauba wax in various ratios and demonstrated that an 85:15 ratio had higher structural properties and oxidative stability properties. Additionally, in our previous research [25], we observed that blending SW and MGs in a 1:1 ratio at a total structurant concentration level of 15% *w*/*w* yielded oleogels that were both stable and significantly harder than those produced from any other tested mixtures of various waxes and MGs; however, the samples were brittle and friable. Hence, the aim of this study was to develop oleogels with SW and MGs, employing low concentrations of structuring agents while, at the same time, maintaining their physical and structural attributes. SW was chosen because it is a highly effective gelling agent capable of providing satisfactory structures even at low concentrations and due to the limited research available on this specific wax. Different total concentrations of either SW or SW combined with MGs were examined to find, for the first time, the optimal combination. To investigate this goal, concentrations of structurants ranging from 6 to 12% *w*/*w* were examined by assessing their textural properties, melting and freezing behavior, and oxidation stability over time. Olive oil was selected due to its high levels of oleic acid and polyunsaturated fatty acids as well as its natural antioxidants such as tocopherols and polyphenols, which contribute to its nutritional value and oxidative stability [43].

## 2. Results and Discussion

### 2.1. Oleogel Appearance

**Figure 1:**
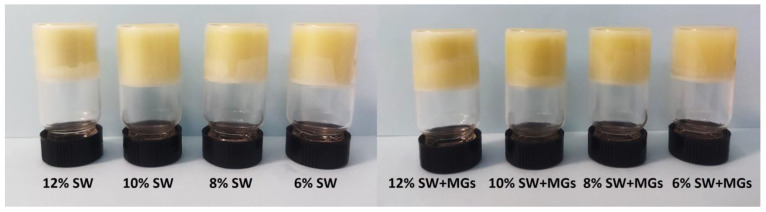
Visual appearance of oleogels created using sunflower wax (SW) and the monoglycerides (MGs) in olive oil.

The visual appearance of olive oil oleogels containing increasing quantities of SW and SW in combination with MGs in a 1:1 ratio is depicted in Figure 1. In all cases, self-standing oleogels were formed, effectively capturing the liquid oil within a solid state.

The color of oleogels is directly affected by the liquid oil as well as the type and concentration of the structural agents used, as demonstrated in the literature [24,25]. Oleogels formed with SW and MGs exhibited high L* values (L*: 72.2–81.0), which increased with the increase in structurants’ concentration (Table 1). The SW samples consistently appeared brighter than those formed by SW and MGs at the same total concentration. Generally, the MGs oleogels had lower L* values compared to the oleogels based on waxes [25], and, therefore, the combination of SW with MGs led to a darker color. 

In all the oleogels, parameter a* had negative values (Table 1), indicating a green hue, as there were no significant differences noted between the samples. Concerning parameter b*, all the oleogels exhibited positive values, suggesting a yellow hue. In most cases, the b* value was higher when using the combination of SW and MGs compared to using solely SW at the same concentration. Overall, the samples displayed hues of greenish-yellow tones, which were directly affected by the olive oil used to form the oleogels [24]. 

### 2.2. Microstructural Assessment

**Figure 2:**
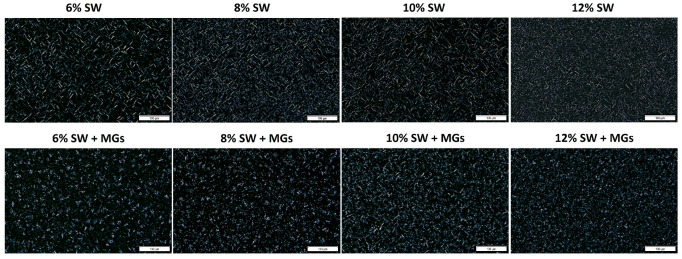
Polarized light micrographs of olive oil oleogels prepared with different concentrations of sunflower wax (SW) and sunflower wax with monoglycerides (SW + MGs) in a 1:1 ratio (scale 100 μm).

The crystal structure of oleogels produced with varying concentrations of SW and SW combined with MGs was investigated through polarized light microscopy after 24 h of oleogel formation (Figure 2). In the oleogel micrographs, the areas with a darker appearance indicate the presence of olive oil, being optically isotropic, whereas the bright areas indicate the presence of crystalline structures. 

Figure 2 depicts SW crystals featuring a long needle-like form, creating highly structured crystal networks capable of trapping considerable oil volumes. The efficiency of wax–oil combinations’ gelling properties is affected by the particular components they contain, specifically, wax esters (96–97%) and free fatty acids (3%) [35,44]. Fayaz et al. [19] pointed out that the presence of the predominant wax ester is associated with the formation of long needle-like crystals. The crystals of SW exhibit a needle-like shape and, as the concentration increases in the system, the crystal network becomes denser and the crystal size decreases. A very similar crystal morphology, long needle-like crystals of SW, was also observed by Doan et al. [45] at a concentration level of 5% using olive oil and by Öğütcü and Yılmaz [46] in soybean oil at a concentration range of 3–10%. 

Oleogels based on MGs exhibit needle-like crystals, characteristic of MG crystallization, as extensively studied in the literature [20,47]. Several researchers have reported that this crystallization type is related to the presence of the β-crystals of MG oleogels [48,49]. The size and the type of crystals can be influenced by a variety of factors, including the concentration of the gelling agent, the type of edible oil used in gel formation, the cooling rate, and the combination with other ingredients [19,50]. However, it is important to note that the addition of SW in combination with MGs as structurants resulted in changes in the crystals’ morphology (Figure 2). As illustrated in Figure 2, the crystals of SW combined with MGs create aggregates, which increase in number and decrease in size with an increasing total concentration of structurants. These results confirm the findings of our previous research [25], which reported that, in the case of combined structurants, the length of the crystals was notably smaller compared to those created by each structuring agent individually, i.e., SW and MGs. Moreover, these results are in agreement with Barroso et al. [23], who observed spherulite crystals to be very closed packed, with the presence of some bigger crystals in the oleogels formed with 6% *w*/*w* SW and MGs at the same concentration in flaxseed oil and stored at 5 °C. The formation of these crystals probably resulted from the interaction between the polar moieties or aliphatic chains of MGs and SW [22,23,36]. 

**Figure 3:**
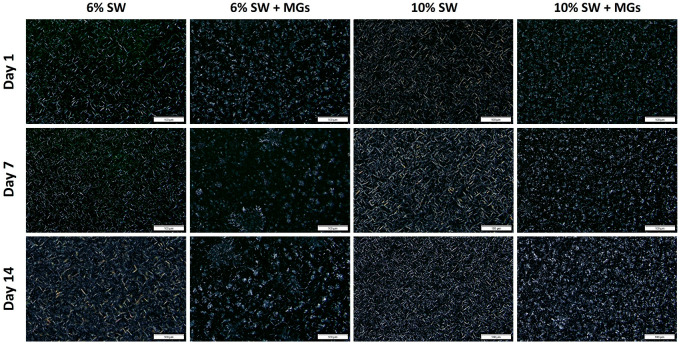
Polarized light micrographs of olive oil oleogels prepared with 6 and 10% (*w*/*w*) of sunflower wax (SW) and sunflower wax with monoglycerides (SW + MGs) in a 1:1 ratio, stored at 5 °C for 1, 7, and 14 days (scale 100 μm).

The crystal morphology of the 6% and 10% oleogels with either SW or both SW and MGs was investigated using polarized light microscopy after 1, 7, and 14 days (Figure 3) of incubation at 5 °C. During the two-week storage period, the oleogels with solely SW did not exhibit any change in crystal type for both of the concentrations studied. These findings align with Doan et al. [45], who did not observe any changes in the crystal type for up to 2 weeks. However, these authors reported that aggregates of spherulite crystals appeared in SW oleogels after three weeks of storage. On the other hand, in the case of SW and MGs oleogels, besides the crystal aggregates, rosette-like crystals were formed, increasing in size and number during the storage period. The alteration in the crystal morphology of oleogels made with SW plus MGs is reflected in the changes observed in the FTIR spectra, as discussed in Section 2.5. The mixed crystals observed in oleogels made with SW and MGs were more prominent at a low gelling agent concentration.

### 2.3. Thermal Analysis

**Figure 4:**
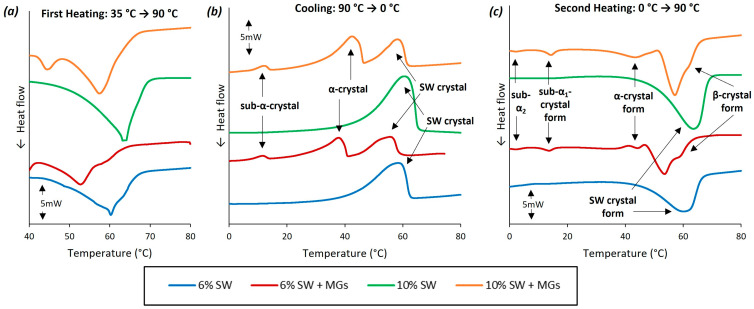
μDSC thermographs of olive oil oleogels prepared with 6 and 10% (*w*/*w*) of sunflower wax (SW) and sunflower wax with monoglycerides (SW + MGs) in a 1:1 ratio; (**a**) first heating run from 30 °C to 100 °C; (**b**) cooling from 100 °C to −10 °C; and (**c**) second heating run from −10 °C to 90 °C.

**Figure 5:**
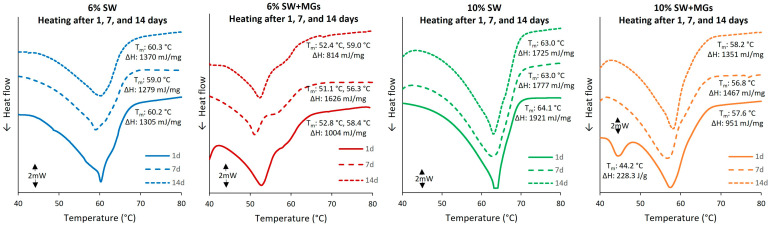
μDSC thermographs of olive oil oleogels prepared with sunflower wax (SW) and sunflower wax with monoglycerides (SW + MGs, in a 1:1 ratio) stored at 5 °C for 1, 7, and 14 days; heating run from 30 °C to 100 °C; heating rate: 1 °C/min.

The characterization of the melting and crystallization behavior of olive oil oleogels, formed with varying concentrations of either SW or combinations of SW and MGs, is depicted in Figure 4 and Figure 5. The oleogels composed solely of SW exhibited a single endothermic peak in the heating process (Figure 4a,c), which shifted to a higher temperature with an increase in the wax concentration. Specifically, the DSC analysis showed that the melting point of 6% SW was 60.2 °C, while, for 10% SW, it was 64.1 °C. Similar trends regarding the effect of wax concentration on melting point values were reported by Martini et al. [51], who studied SW, paraffin wax, and beeswax at different concentrations, i.e., 1–10%, using several types of oils. In general, SW is mainly composed of a significant quantity of long-chain wax esters (96–97%, mainly C22–24), a small fraction of free fatty acids (3%, C16–22), and trace amounts of free fatty alcohols and hydrocarbons [35,44]. Therefore, the high melting temperature of SW oleogels is a result of the significant amount of long-chain wax esters. As depicted in Figure 4a, a minor shoulder was observed subsequent to the main endothermic peak, associated with wax esters, indicating the melting of additional molecular components, likely the free fatty acids [45]. This shoulder vanished during crystallization (Figure 4b) and the second heating run (Figure 4c), creating a broader crystallization/melting peak, which indicated an overlapping melting point with the dominant wax ester fraction. This shoulder was detected solely at a 6% SW concentration, likely attributed to the low wax concentration in the oleogel, coupled with the high precision of the μDSC instrument and the low heating rate (1 °C/min).

The μDSC analysis demonstrated that the combination of SW with MGs in a 1:1 ratio led to two endothermic peaks during the initial heating phase, three exothermic peaks in the cooling run, and five endothermic peaks during the second heating cycle. Generally, as the quantity of added wax and monoglycerides increased, the melting/crystallization temperatures and enthalpies also increased [10,36,52]. Regarding the first heating run, the endothermic peak was observed for 6% and 10% SW + MGs oleogels at 52.8 and 57.6 °C, respectively. In the case of 6% SW + MGs, a small shoulder was detected at 58.4 °C, which was likely associated with the melting of monoglycerides. However, this shoulder was not distinct in the case of 10% SW + MGs, resulting in a broader peak compared to either SW or MGs, as described above. The resulting melting points observed in this study were in agreement with those documented in the existing literature. Specifically, Öğütcü and Yılmaz [46] stated that the melting point of oleogel formed with 3% SW was 58.4 °C, while Hwang et al. [52] reported that the melting point of oleogels formed with 0.5–10% SW in soybean oil ranged from 47.0 to 65.0 °C.

The crystallization profile of oleogels showed a broad exothermic peak at 58.3 and 60.5 °C for 6% and 10% SW, while, in the case of 6% and 10% SW + MGs oleogels, the first exothermic peaks were noted at 55.6 and 58.3 °C, respectively. As evident from the results, the concentrations of 6% SW and 10% SW + MGs, which had, approximately, the same amount of wax, exhibited crystallization at the same temperature. This implied that the observed peak was associated with the crystallization of wax. The same melting temperature were reported by Doan et al. [45], who concluded that the oleogels with 5% SW in rice bran oil had a temperature for crystallization of 60.4 °C and a temperature for melting of 62.8 °C, and, as the concentration decreased, the crystallization and melting temperatures also decreased. Concerning the samples containing wax and monoglycerides, they displayed a second exothermic peak at 37.8 and 42.4 °C and a third at 11.5 and 12.0 °C for 6% and 10% SW + MGs, respectively. These peaks were associated with the structures of monoglycerides and corresponded to α- and sub-α crystals, respectively [47,48,53]. Specifically, when MGs were dissolved in oil (at a high temperature) and the system was cooled at room temperature, an inverted lamellar state, an α-crystal network, was formed. Upon further cooling to 5.0 °C, a part of the α-crystal network was transformed to sub-α; this transition temperature corresponded to the crystallization of fatty acid aliphatic chains in the lamella [54]. Hence, the temperature peak at approximately 40.0 °C was linked to α-crystals, while the peak ranging from 11.5 to 12.0 °C was linked to sub-α crystals [8,47,53]. 

Upon the second heating cycle, the oleogels with pure SW showed a single endothermic peak at 60.4 and 63.6 °C for 6 and 10% SW, respectively, values corresponding to those observed in the initial heating cycle. On the other hand, five peaks were present for the oleogels consisting of SW and MGs, corresponding to sub-α_2_, sub-α_1_, α-, and β-crystal forms [47,53] and SW crystals. The sub-α_2_ crystals formed at 2.2 °C [49], while the sub-α_1_ crystals formed at 13.7 and 14.4 °C [53] for 6 and 10% SW + MGs, respectively. The α-crystals of MGs exhibited a peak at around 44 °C, while the endothermic peaks attributed to the SW crystals were at 53.6 and 57.2 °C for 6 and 10% SW + MGs, respectively. In addition, as illustrated in Figure 4, a shoulder was detected at 58.5 and 65.0 °C for oleogels formed with 6 and 10% SW + MGs, which was attributed to the β-crystal forms. Regarding the melting enthalpy values, the SW + MGs oleogels showed lower ΔH values in comparison to the oleogels formulated exclusively with SW at the same total concentration. Moreover, higher ΔH values were noted as the concentration of gelling agents increased. In general, lower melting enthalpies suggest reduced energy requirements for the organization of the crystalline network. Therefore, for the same concentration of gelators, SW + MGs require a lower energy to form the crystalline network, which is also harder, according to measurements from the texture analysis presented in Section 2.4. 

When stored at 5 °C, there were no observable changes in the melting temperatures and melting enthalpies for oleogels containing solely SW. These findings were in agreement with Doan et al. [45], who noted that the storage of oleogels for 4 weeks at 5 °C did not affect the melting temperature of the primary peak of 5% SW (62.7 °C). Instead, after 4 weeks, they observed a new peak at around 21.0 °C, attributing it to the formation of a new crystalline form, i.e., spherulite clusters. Additionally, Zampouni et al. [20] reported that the melting point of oleogels with 15% MGs and 15% MGs plus 5% phytosterols did not change after 14 days at different storage temperatures. Regarding the oleogels containing SW and MGs, a distinction was observed between the first day and after 7 days (and 14 days) of storage, as the endothermic peak at around 40.0–50.0 °C disappeared. This peak was associated with the α-crystal network, considered a metastable phase, which transformed into the more thermodynamically stable β-crystals during a storage period of approximately two to three days [8,49,54]. This transformation was further supported by the melting enthalpies of the peaks, evident in the case of 10% SW + MGs (Figure 5); after 7 days, the melting enthalpy was the sum of the enthalpies of the two peaks observed on the first day.

### 2.4. Texture Analysis

Texture properties, particularly hardness, are essential functional aspects of oleogels that determine their suitability for use in various food products [19,36,46]. In the oleogels formed by both plain SW and a combination of SW and MGs, the hardness increased proportionally with the concentration of the structurants (Table 1). This finding was in agreement with the conclusions drawn by Hwang et al. [55] and Öğütcü and Yılmaz [46], who observed increased hardness values with the increase in SW concentration, ranging from 3% to 7% in soybean oil and from 3 to 10% in hazelnut oil, respectively.

The oleogels formed by combining MGs with SW led to a different crystal structure, directly affecting the textural properties of the oleogels. Specifically, at the concentration level of 10 and 12%, the hardness of the mixed-component oleogels was 25–30% greater compared to those formed solely by SW, while, at lower concentrations (6–8%), the opposite behavior was observed. We believe that the difference in this behavior is related to the concentration of the components; that is, monoglycerides in a low concentration are not sufficient to strengthen the crystalline network, and they eventually weaken it by disrupting SW crystallization. Based on the texture analysis results, less than 4% MGs (8% total structurants) weaken the crystalline network formed by SW, whereas more than 4% MGs strengthen the SW–MGs network. Above this critical concentration, the combination of SW and MGs gives a higher hardness compared to the same concentration consisting only of wax. Moreover, as depicted in Figure 2, in the MGs and SW oleogels containing >8% *w*/*w* (4% SW and 4% MGs), the length of the crystals and aggregates was much smaller, and, therefore, the network was firmer than the oleogels with 6% and 8% SW and MGs. These results align with our previous study, where the concentration of structurants remained constant at 15% and it appeared that the combination of SW with MGs in a ratio 1:1 gave a higher hardness than the oleogels from the individual components [25]. 

### 2.5. FTIR Analysis

FTIR spectroscopy was used in this study in order to provide insights into the intermolecular interactions and the chemical groups that significantly contributed to the development of the oleogel network. 

**Figure 6:**
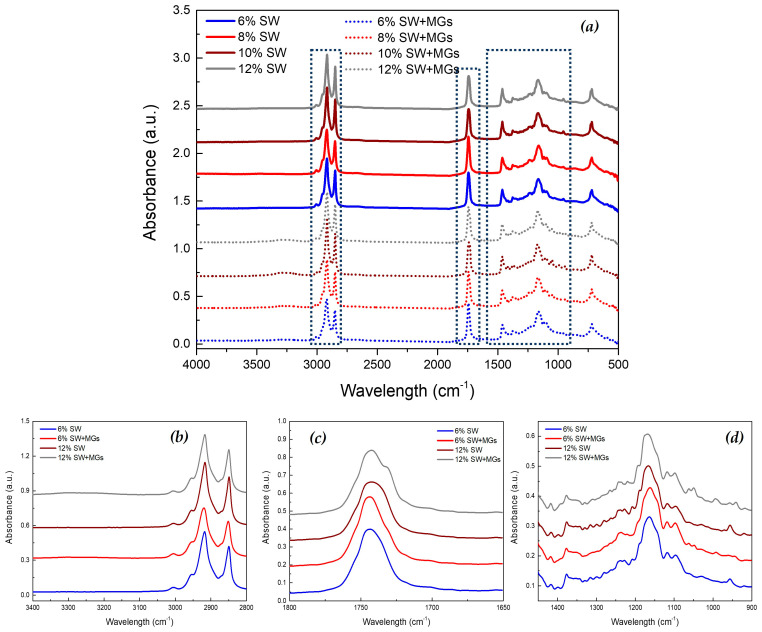
FTIR spectra of olive oil oleogels created with a variety of concentrations of sunflower wax (SW) or sunflower wax with monoglycerides (SW + MGs) in a 1:1 ratio (spectral regions: (**a**) 4000–500 cm^−1^, (**b**) 3400–2800 cm^−1^, (**c**) 1800–1650 cm^−1^, and (**d**) 1450–900 cm^−1^).

The pure MGs and the oleogels containing MGs showed a broad double peak in the region of 3100–3400 cm^−1^ (at ~3240 and ~3306 cm^−1^), which corresponded to hydrogen bonding between the 2-OH and 3-OH of polar groups [36,48]. These hydrogen bonds are generally associated with the formation of the inverse lamellar phase and the creation of the stable β-crystal polymorph [20,48], and, therefore, they contribute to the establishment of the crystal network in an oleogel’ formation [47]. On the contrary, in solely SW-based oleogels, these two peaks were absent because the formation of such hydrogen bonds did not occur. In the oleogels containing both SW and MGs, as the concentration of MGs in the system increased, these peaks became more intense, exhibiting increased absorbance (Figure 6). However, the variances in the absorbance of the peaks were relatively small and are not clearly shown in Figure 6. These results concerning the two peaks were in agreement with Öğütcü and Yılmaz [37], who observed the presence of weak intermolecular -OH hydrogen bonds exclusively in MG oleogels because of the existence of hydroxyl groups, while such bonds were not observed in oleogels with carnauba wax.

The two absorbance peaks observed at ~2917 and ~2850 cm^−1^ were related to the C–H stretching vibration [48], and the intensity was higher in pure SW-based oleogels because waxes contain a long-chain alkane backbone [56]. These two peaks are related to the van der Waals interactions between long alkyl chains or fatty acid tails, and, therefore, affect the formation of the oleogel network [36].

The absorbance peak at 1743 cm^−1^ associated with the stretching vibration of the carbonyl group C=O [57] was noted in all the samples. In the case of oleogels formed with MGs and SW, the peak intensity was higher compared to the oleogels with SW at the same total concentration, while a shoulder at ~1730 cm^−1^ was observed (Figure 6). The peak at ~1730 cm^−1^ had also been reported in our previous study [25]. This peak is characteristic for MGs, and it decreased as the concentration of MGs in the system decreased. This peak corresponds to the carbonyl group of the ester bond between glycerol and fatty acids [20]. The same observation was mentioned by Pakseresht et al. [36], who reported that the peak intensity was more pronounced in MGs compared to carnauba wax and decreased as the concentration of wax in the system increased.

The absorbance peaks in the range of 1000–1300 cm^−1^ are mainly related to C–O stretching vibrations in the ester group and C–H bending vibrations [25,47]. Moreover, the peaks at ~1345 cm^−1^ and 1453 cm^−1^ correspond to the stretching vibration of C–H in the CH_2_ and CH_3_ groups [58]. These two peaks signify the van der Waals interactions of the adjacent aliphatic tails of MGs and the long alkyl chains of waxes [47]. Additionally, there are differences in the spectra of different oleogels, suggesting disparities in the chemical composition between SW and MGs. Specifically, a peak at 1179 cm^−1^ represents the stretching vibration of non-hydrogen-bonds of C–OH groups, a peak at 1161 cm^−1^ relates to the stretching vibration of the C–OH groups involved in hydrogen bonding, and peaks at 1048 and 1062 cm^−1^ correspond to the stretching vibration of C–O bonds [47,58]. These peaks are shown only in oleogels containing MGs, and they might indicate the organization of hydrophilic groups in the crystal network. The absorbance peak at 940 cm^−1^ denotes the bending vibration of hydrogen bonds between carboxylic acids. This peak was observed for MGs and oleogels with MGs [57], while the peak was absent in the SW oleogels [36]. 

**Figure 7:**
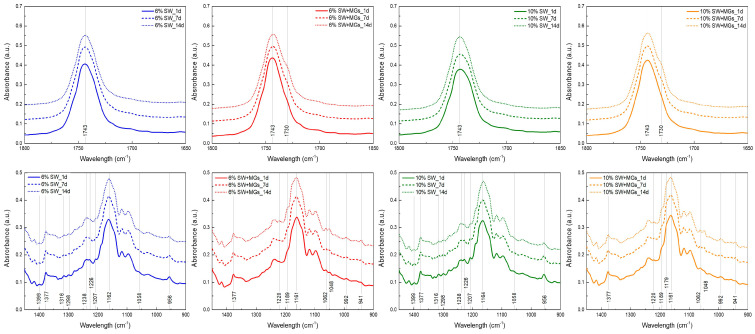
FTIR spectra of olive oil oleogels, prepared with 6 and 10% (*w*/*w*) of sunflower wax (SW) and sunflower wax with monoglycerides (SW + MGs) in a 1:1 ratio, stored at 5 °C for 1, 7, and 14 days (first raw: spectral region 1800–1650 cm^−1^, second raw: spectral region 1450–900 cm^−1^).

The FTIR spectra of oleogels stored at 5 °C for two weeks are depicted in Figure 7. In the case of the oleogels containing only SW, the spectra showed no significant variations during their storage for 14 days. There were very slight differences at 956, 1058, 1164, 1207, and 1226 cm^−1^, but they were minimal. These findings were aligned with the observed crystals and DSC results, as the wax crystals maintained their form without undergoing changes during the storage period. In the case of oleogels containing MGs, the absorbance peak at ~3230 cm^−1^ became more distinct during the storage periods of 7 and 14 days. According to the literature [20,59], the formation of this peak is linked to the transition from α- to β-crystals. Moreover, some modifications in terms of peak creation and intensity were shown at 1393, 1178, 1193, 1178, 1062, 1048, and 942 cm^−1^, which may have been related to the changes in crystal morphology observed by microscopy. 

These observations led to the hypothesis that, in oleogels containing MGs with SW, hydrogen bonding interactions are probably the primary mechanism enhancing the physical attributes of the oleogels, followed by van der Waals interactions. On the other hand, in SW oleogels, gel formation mainly arises from the synergistic effects of van der Waals forces, crystal morphology, and their spatial arrangement [60].

### 2.6. Oxidative Stability

**Figure 8:**
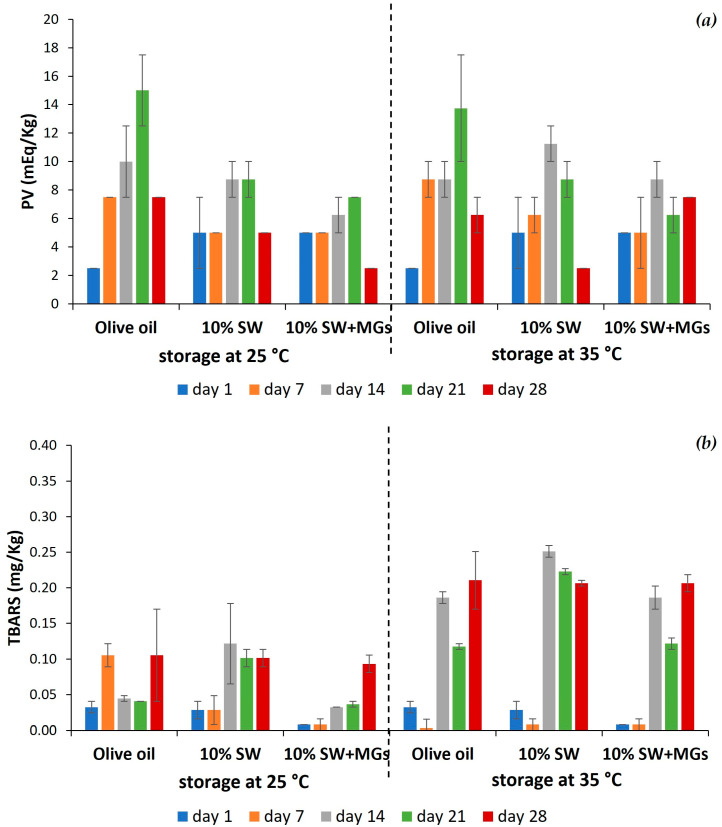
Evolution of peroxide (**a**) and TBARS (**b**) values in liquid olive oil, SW-based oleogels, and SW plus MGs-based oleogels during storage at 25 and 35 °C over time.

Lipid oxidation occurs in three phases, namely, initiation, propagation, and termination. During this process, in the initiation and propagation stages, primary oxidation products, such as hyperoxides, are formed, which subsequently react with fatty acids and other substances and form the secondary oxidation products, like aldehydes, ketones, alcohols, and hydrocarbons [61,62]. Lipid oxidation can also alter fatty acid composition due to the formation of trans and conjugated double bonds. The products formed during lipid oxidation lead to the development of an unacceptable flavor, and, furthermore, some of these oxidation products are toxic [62]. In order to determine the lipid oxidation of olive oil oleogels, peroxide values (PV), which measure the primary oxidation products, and thiobarbituric acid-reactive substances (TBARS), corresponding to the secondary oxidation products, were examined. The storage of the samples was performed at two high temperatures, 25 and 35 °C, in order to accelerate lipid oxidation and monitor their oxidative stability. The changes in the PV and TBARS of the olive oil oleogels created by either 10% SW or 10% SW + MGs as well as in the values of liquid olive oil, which was heated simulating the processing conditions of the oleogels, during the storage period of 28 days are depicted in Figure 8.

The PV of the heated olive oil were consistently higher throughout the storage period compared to those of the oleogels, except for the first day (Figure 8a). These results indicate that oleogels, which entrapped the oil in a semi-solid structure, resulted in an increased resistance to oxidation compared to liquid olive oil, and, therefore, the samples were more stable to deterioration. Moreover, in most cases, the PV of the samples stored at 35 °C were slightly higher than those stored at ambient temperature, without being statistically significant. These results are in line with the literature, as the higher temperature favors the formation of radicals, which are responsible for the initiation and propagation of lipid oxidation [63]. These notions are aligned with the work of Samui et al. [62], who reported that the oxidation rate of canola oil was higher than that of oleogels, formulated using glycerol monostearate and lecithin, throughout a storage period of 30 days at different storage temperatures, that is, 4, 25, and 40 °C.

The PV of the oleogels increased over time, with a more pronounced increase being noted in the samples stored at 35 °C (*p* > 0.05). Concerning oleogel composition, it appears that the oleogels formed solely with SW exhibited slightly higher PV than those containing SW and monoglycerides, although these differences were not statistically significant. It is worth noting that the PV of the oleogels throughout the storage period of 4 weeks were below the upper limit, which is 10 mEq/kg for oil, according to the Codex standard for edible fats and oils [64]. In general, PV in the range of 1 to 5 mEq/kg indicate a low level of oxidation development, values between 5 and 10 mEq/kg are considered to indicate a moderate level of oxidation, whereas those exceeding 10 mEq/kg signify a high level of oxidation [65]. According to the above, the oleogels in our study exhibited oxidative stability when stored for 4 weeks at 25 and 35 °C. These results are in agreement with Öğütcü and Yılmaz [46], who mentioned that the oleogels formed with SW and carnauba wax in hazelnut oil were very stable against oxidation during three months of storage at 4 and 20 °C. Additionally, Hwang et al. [66] observed that the oxidative stability of oleogels is affected by the wax type and the storage temperature. Specifically, the authors studied the oxidative stability of fish oil oleogels formed with rice bran wax, sunflower wax, candelilla wax, and beeswax. On the contrary, Orhan and Eroglu [67] showed higher PV for oleogels prepared with beeswax, SW, and carnauba wax in black cumin oil during a storage period of eight weeks. The decrease in PV observed on the 28th day was expected, as hydroperoxides, which are the primary oxidation products, further oxidize to aldehydes and ketones, which are the secondary oxidation products, measured as TBARS.

The TBARS values of the oleogels during a storage period of 28 days are illustrated in Figure 8b. The oleogels, prepared with SW, or a combination of SW and MGs, and liquid oil had very low TBARS values throughout the storage period. An increase was noted for all the samples stored at 35 °C compared to those stored at 25 °C, while the values increased over time. Similar, low TBARS values were observed by Samui et al. [62] for oleogels composed of glycerol monostearate and lecithin in canola oil, and they were stored at 25 °C.

Considering both the PV and the TBARS values, the oleogels formulated with either SW or SW and MGs showed oxidative stability for at least one month at ambient temperature. This suggests that a further extension of their shelf life could be achieved by storing them at refrigerated temperatures.

## 3. Conclusions

In this study, olive oil oleogels created by either pure SW or a combination of SW and MGs in a 1:1 ratio, at different concentrations, were examined, and the synergistic behavior of the two components was assessed. All the oleogels remained shelf-stable at ambient temperature, with increased hardness being observed as the total concentration of gelators increased. Furthermore, a positive interaction between SW and MGs was observed above a 4% *w*/*w* concentration of MGs, contributing to the increased hardness of the oleogels. The crystals of the SW oleogels were long and needle-like, while the combination of SW and MGs resulted in the formation of crystal aggregates and rosette-like crystals. The microscope images revealed changes in the crystalline structure due to the use of different gelling agents and during storage at refrigeration temperatures for 2 weeks. These changes were further confirmed by the results of the DSC and FTIR analyses. The findings suggested that hydrogen bonding and van der Waals forces predominantly governed the SW and MGs oleogels, while van der Waals interactions were responsible for the SW oleogels. Additionally, the oleogels formulated with SW and a combination of SW plus MGs exhibited a high stability in terms of oxidative stability during a 4-week storage period at ambient temperatures. Therefore, we can conclude that using a blend of two economic gelling agents provides several benefits, including a reduction in the overall concentration of gelling agents and the avoidance of a pronounced waxy mouthfeel. Hence, the developed oleogels could serve as an alternative hard fat source in various food products once SW receives regulatory approval. Furthermore, in this research, the MGs used were mostly saturated palmitic acid (C16:0); however, if other MGs are used, i.e., C14:0 or C18:0, the properties of the oleogels would slightly be changed, enabling them to be used in different applications. Further research into the application of these structures in real foods and their behavior during product formation would provide clarity regarding their appropriate usage.

## 4. Materials and Methods

### 4.1. Raw Materials and Oleogels’ Preparation

Sunflower wax (SW) (Daraveli and Co., Ltd., Athens, Greece) and monoglycerides (MGs) (HARI 95 Riketa SDN BHD, Johor Bahru, Malaysia) were employed in the formulation of different oleogels. The MGs exhibited at least a 95% monoester content, with the acid value not exceeding 3%, the iodine value being capped at 2%, and the free glycerin value limited to 1%. The MGs were derived from hydrogenated palm oil, with palmitic acid being the main fatty acid (C16:0). According to the manufacturer’s specifications, SW presented an acid value of 2.3 mg KOH/g and a saponification value of 91.6 mg KOH/g. The melting points for the structuring agents were reported to be 75 °C and 71 °C, for SW and MGs, respectively.

Different amounts of SW (6, 8, 10, and 12% *w*/*w*) or a mixture of SW and MGs in a 1:1 ratio (6, 8, 10, and 12% *w*/*w*) were studied in order to form shelf-stable oleogels. For oleogel preparation, appropriate quantities of olive oil (Minerva SA, Metamorphosi, Greece) were weighed and preheated at 85 °C with continuous stirring. Following that, the structuring agents were gradually added, fully dissolved, and the mixtures underwent stirring for 30 min at a temperature of 90–95 °C [21,25]. The resulting liquified oleogels were subsequently transferred into plastic containers (polypropylene, 10 × 10 cm), followed by cooling at room temperature for 40 min. Following this, the oleogels were stored at 5 °C for 1, 7, and 14 days. The measurements were carried out at room temperature, with the oleogel samples first being allowed to reach equilibrium at 25 °C for 3 h. All the measurements were conducted in duplicate using distinct batches of oil.

### 4.2. Color Measurement

Color measurements were conducted using a Chroma Meter CR-400 (Minolta, Osaka, Japan), utilizing a D 65 light source. The color of the oleogels was assessed by recording the L* (brightness), a* (+/−, red-to-green spectrum), and b* (+/−, yellow-to-blue spectrum) values after calibrating the colorimeter with a supplied white tile. The oleogels samples were prepared as descripted previously, stored at 5 °C for 24 h, and then cut into cubes of 20 mm, following 3 h equilibration at 25 °C. For each oleogel sample, ten measurements were obtained from the surface of the cubes, with the outcomes being reported as the mean ± standard deviation.

### 4.3. Polarized Optical Microscopy

The microstructural images of the oleogels were captured using an Olympus BX43 polarizing microscope (Olympus Optical Co Ltd., Tokyo, Japan), which was equipped with a digital microscope camera (Basler USB3 Vision, Ahrensburg, Germany). Specifically, one drop of liquified oleogels was placed on a preheated microscope slide and was covered by a glass coverslip. Subsequently, the samples were cooled at 5 °C for the predetermined period mentioned earlier. The morphological characteristics were noted at 25 °C, employing a 20× magnification objective (NA 0.75), and the images of the oleogels were captured utilizing the Basler Microscopy Software (version 2.1) (Basler, Ahrensburg, Germany).

### 4.4. Differential Scanning Calorimetry (DSC)

The investigation of the melting and crystallization behaviors of the oleogels was conducted using a microcalorimeter (microCalvet μDSC 7 Evo-1A, Setaram, Caluire-et-Cuire, France), equipped with two parallelly positioned 1 mL capsules. One capsule contained the sample, while the other contained olive oil and was used as a reference sample. The samples were heated from 30 °C to 100 °C (first heating run), remained at this temperature for 5 min to erase the crystal memory of the samples, and then the oleogels were cooled to −10 °C and remained at this temperature for 5 min. Afterwards, the samples were reheated to 90 °C (second heating run). Throughout the measurements, there was a continuous flow of nitrogen gas (0.8 bar). The heating and cooling rate was set to ±1 °C/min. The sample quantity was 400 ± 10 mg. The μDSC was calibrated using a standard sample of pure naphthalene. The peak melting temperature (Tm), peak crystallization temperature (Tc), and apparent melting/crystallization enthalpy (ΔH) of the structured emulsions were defined from the endothermic and exothermic peaks of the μDSC scans, respectively, using the CALISTO v.2.14 software (Setaram, Caluire-et-Cuire, France). The measurements were carried out one, seven, and fourteen days after the oleogels’ preparation. All the μDSC experiments were conducted in triplicate.

### 4.5. Texture Analysis

The texture properties of the olive oil oleogels were evaluated by means of texture profile analysis (TPA), employing a double compression cycle test [21] with a Universal TA.XT plus Texture Analyzer (Stable Micro Systems, Godalming, Surrey, UK) equipped with a 5 kg load cell. Following 3 h equilibration at 25 °C, the samples were cut into cubes of 20 mm. The test speed was set to 1.0 mm/s, compressing the samples to 75% of their initial height using an SMS P/100 probe (with a plate probe diameter of 100 mm). The TPA parameters assessed were hardness (N) (the maximum force required during the initial compression) and cohesiveness (the ratio of the two areas under the first and second compression curves). Each treatment was subjected to six assessments, with the outcomes being presented as the mean ± standard deviation.

### 4.6. Fourier Transform Infrared Spectroscopy (FTIR)

The FTIR spectra of the oleogels were acquired using an FTIR 6700 series spectrometer (JASCO, Japan) equipped with three-reflection ATR diamond (MIRacle ATR, Pike Technologies, Madison, WI, USA). Following 3 h equilibration at 25 °C, the oleogel samples were placed on the ATR plate, and the spectra were recorded across a wavenumber range of 4000 to 500 cm^−1^, with a spectral resolution of 4 cm^−1^, and 32 scans. A background spectrum of air was captured and subtracted from each sample’s spectrum before the measurements. For each treatment, five spectra were acquired, and the average of these spectra was used for the analysis. The analysis of the spectra was carried out using Spectra manager (v.2, Jasco, Tokyo, Japan).

### 4.7. Oxidative Stability of Oleogels

For the assessment of oxidative stability, the oleogels were stored at room temperature (25 °C) and at 35 °C in order to accelerate lipid oxidation. The oxidative stability of the oleogels was assessed by determining the primary oxidation products, i.e., the peroxide values (PV), and the secondary oxidation products, i.e., the thiobarbituric acid-reactive substances (TBARS). The measurements were performed on 1, 7, 14, 21, and 28 days.

The PV were assessed following the AOAC Official Method 965.33, with minor adjustments. In summary, 5 g of sample was combined in an iodine flask with 25 mL of a 3:2 acetic acid–chloroform solution and 1 mL of saturated KI solution. Then, the mixture was shaken and left in dark conditions for 1 min. Following that, 75 mL distilled water and 2 mL starch indicator (1% *w*/*w*) were added, and the solution was titrated against 0.05 N sodium thiosulphate solution until the disappearance of the blue color. At the same time, a blank test was measured without the oleogel sample. The PV were calculated with the equation provided below:

(1) $$PV=\frac{N\cdot (V-V_{0})}{w}\cdot 1000$$

where *N* is the normality of the sodium thiosulphate solution, *V* and *V*_0_ are the volume (ml) of the sodium thiosulphate solution used for the titration of the samples and the blank test, respectively, and *w* is the samples’ weight (g). The determination of the PV was performed in duplicate, and the results were expressed as mEq/Kg.

The TBARS were assessed following the method described by Katsanidis and Zampouni [68] through the steam distillation process. The TBARS concentration was measured by absorbance measurements at 532 nm (UV-1700 spectrophotometer, Shimadzu Europe GmbH, Duisburg, Germany). Duplicate samples were examined, and the results were presented as mg of the malonaldehyde (MA)/kg of the sample.

### 4.8. Statistical Analysis

The gathered data underwent analysis through ANOVA, employing the general linear model with a significance threshold set to α = 0.05. To discern differences between the treatments, Tukey’s test was applied. This statistical evaluation was executed utilizing the MINITAB v. 16 software (Minitab, Inc., State College, PA, USA).

## Figures and Tables

**Table 1:** Impact of structural agent on physicochemical and color parameters of oleogels.

Sample	Physicochemical Parameters		Color Parameters		
	Hardness	Cohesiveness	L*	a*	b*
6% SW	7.36 ± 0.42 ^e,f^	0.168 ± 0.013 ^a,b^	76.1 ± 1.1 ^d^	−8.9 ± 0.1 ^a^	30.9 ± 0.6 ^a^
6% [SW + MGs]	3.51 ± 0.24 ^f^	0.182 ± 0.025 ^a^	72.2 ± 1.2 ^e^	−7.8 ± 0.2 ^a^	28.2 ± 0.7 ^c^
8% SW	13.27 ± 0.65 ^c,d,e^	0.091 ± 0.003 ^b,c^	76.4 ± 1.4 ^d^	−8.5 ± 0.2 ^a^	27.6 ± 0.8 ^c,d^
8% [SW + MGs]	10.20 ± 1.69 ^d,e,f^	0.095 ± 0.026 ^b,c^	75.8 ± 1.0 ^c,d^	−8.3 ± 1.7 ^a^	30.0 ± 0.5 ^a,b^
10% SW	15.32 ± 0.77 ^c,d^	0.081 ± 0.010 ^c^	80.0 ± 0.9 ^a,b^	−8.4 ± 0.1 ^a^	26.8 ± 0.5 ^d,e^
10% [SW + MGs]	19.98 ± 2.5 ^c^	0.043 ± 0.012 ^c^	77.2 ± 2.2 ^c,d^	−8.6 ± 0.2 ^a^	27.1 ± 0.8 ^d,e^
12% SW	27.32 ± 1.45 ^b^	0.070 ± 0.012 ^c^	81.0 ± 0.9 ^a^	−8.3 ± 0.1 ^a^	26.6 ± 0.3 ^e^
12% [SW + MGs]	35.74 ± 0.09 ^a^	0.024 ± 0.001 ^c^	78.7 ± 1.4 ^b,c^	−9.1 ± 0.2 ^a^	29.5 ± 0.9 ^b^

* Data are presented as the means ± standard errors. Means with different superscript letters for the same parameter are significantly different (*p* < 0.05).

## Data Availability

The data presented in this study are available upon request from the corresponding author.
